# Preclinical biodistribution, tropism, and efficacy of oligotropic AAV/Olig001 in a mouse model of congenital white matter disease

**DOI:** 10.1016/j.omtm.2021.01.009

**Published:** 2021-01-21

**Authors:** Jeremy S. Francis, Vladimir Markov, Irenuez D. Wojtas, Steve Gray, Thomas McCown, R. Jude Samulski, Marciano Figueroa, Paola Leone

**Affiliations:** 1 Cell and Gene Therapy Center, Department of Cell Biology & Neuroscience, Rowan University School of Osteopathic Medicine, Stratford, NJ, USA; 2 Department of Pediatrics, The University of Texas Southwestern Medical Center, Dallas, TX, USA; 3 Gene Therapy Center, Department of Psychiatry, University of North Carolina, Chapel Hill, NC, USA; 4 Asklepios BioPharmaceutical, Research Triangle Park, NC, USA

## Abstract

Recent advances in adeno-associated viral (AAV) capsid variants with novel oligotropism require validation in models of disease in order to be viable candidates for white matter disease gene therapy. We present here an assessment of the biodistribution, tropism, and efficacy of a novel AAV capsid variant (AAV/ Olig001) in a model of Canavan disease. We first define a combination of dose and route of administration of an AAV/Olig001-GFP reporter conducive to widespread CNS oligodendrocyte transduction in acutely symptomatic animals that model the Canavan brain at time of diagnosis. Administration of AAV/Olig001-GFP resulted in >70% oligotropism in all regions of interest except the cerebellum without the need for lineage-specific expression elements. Intracerebroventricular infusion into the cerebrospinal fluid (CSF) was identified as the most appropriate route of administration and employed for delivery of an AAV/Olig001 vector to reconstitute oligodendroglial aspartoacylase (ASPA) in adult Canavan mice, which resulted in a dose-dependent rescue of ASPA activity, motor function, and a near-total reduction in vacuolation. A head-to-head efficacy comparison with astrogliotropic AAV9 highlighted a significant advantage conferred by oligotropic AAV/Olig001 that was independent of overall transduction efficiency. These results support the continued development of AAV/Olig001 for advancement to clinical application to white matter disease.

## Graphical Abstract

**Figure:**
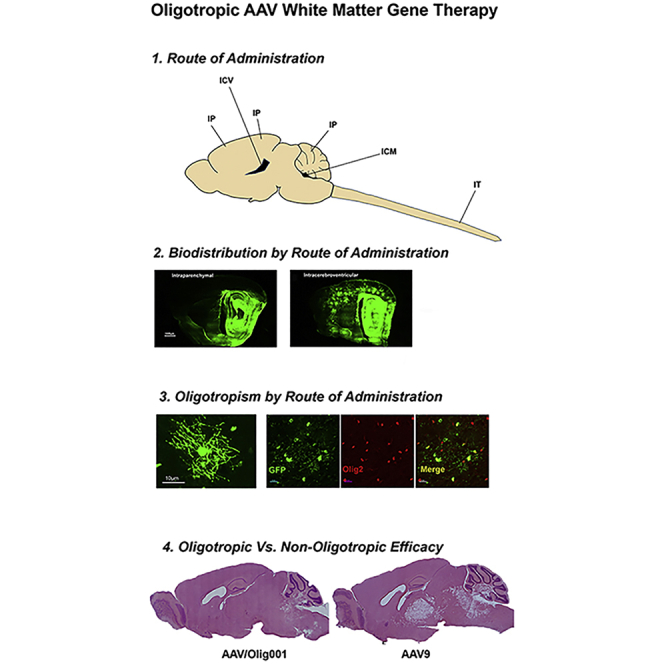

## Teaser

This study documents the unique target engagement features of a novel viral vector (AAV/Olig001) that promotes phenotypic rescue in a preclinical model of a white matter disease caused by an inherited defect in a white matter cell enzyme.

## Introduction

Recombinant adeno-associated viral (AAV) vectors present a combination of safety and efficacy that is attractive to clinical gene therapy in the brain. AAV has been used in clinical trials for a broad spectrum of CNS diseases, but oligodendrocytes remain somewhat refractory to gene delivery by available serotypes, thereby restricting potential for white matter diseases. Although it is possible to utilize promoter elements that limit transgene expression to specific cellular lineages, the capsid-cell surface interaction is the primary determinant of AAV tropism.^1^^,^^2^ Most native AAV serotypes are inherently neurotropic in the main, but engineered capsid variants have the potential to expand the tropic repertoire of AAV independently of restrictive transcriptional machinery. A recent development in this context is a novel AAV variant capable of efficient transduction of CNS white matter-producing cells.^3^ This engineered variant, AAV/Olig001, utilizes a capsid with preferential tropism for the oligodendrocyte cell surface, meaning strong constitutive promoters can be employed for transgene expression in order to achieve maximal effect in the target cell lineage. We have previously shown AAV/Olig001 capable of transducing neonatal oligodendrocytes in a mouse model of the congenital leukodystrophy Canavan disease, resulting in rescue of the congenital genetic defect and resistance to disease.^4^ This important proof of concept demonstrates the ability of AAV/Olig001 to reconstitute white matter-specific enzyme function^5^^,^^6^ in its natural lineage compartment. In humans, Canavan disease manifests in early postnatal life, with diagnosis typically occurring after key neurodevelopmental milestones have been missed,^7^ thereby defining patients in need of therapy as symptomatic. Our previously published study documenting correction of the Canavan genetic defect in a mouse model^4^ used AAV/Olig001 to deliver a therapeutic transgene in presymptomatic neonatal animals, prior to the presentation of severe motor and histopathological abnormalities. If AAV/Olig001 is to realize its clinical potential, then it must be shown capable of maintaining this inherent specificity for oligodendrocytes in an acutely symptomatic microenvironment. A very limited number of studies have shown the ability of AAV/Olig001 to transduce oligodendrocytes *in vivo* in healthy rodents^3^^,^^8^ and nonhuman primates^8^ with upward of 90% oligotropism. AAV/Olig001 has been shown to partially rescue the phenotype in a mouse model of lysosomal storage disease,^9^, but no comprehensive analysis of the potential therapeutic benefits of AAV/Olig001 tropism has been undertaken in a bona fide model of symptomatic congenital white matter disease.

The present study addresses the need for validation of AAV/Olig001 oligotropism in this context by way of a comprehensive assessment of biodistribution and tropism in the nur7 mouse model of Canavan disease. Canavan disease is caused by inherited mutations in the gene encoding for the enzyme aspartoacylase (ASPA)^10^ which results in the accumulation of its substrate, N-acetylaspartate (NAA). Pathologically elevated NAA with associated loss of ASPA activity is the primary diagnostic hallmark of Canavan disease. The nur7 mouse is an (ENU)-induced mutant that has a nonsense point mutation in the *ASPA* gene, resulting in the absence of functional protein and chronically elevated CNS NAA.^11^ nur7 pathology is progressive, with deteriorating motor function and CNS cell loss manifest from 2 to 3 weeks of age onward. This age threshold distinguishes presymptomatic from acutely symptomatic, with the two contexts offering markedly distinct environments that potentially influence the biodistribution and tropism of specific gene-delivery systems. nur7 mice present evidence of attempted remyelination in the brain in the form of increased markers of immature oligodendrocytes as late as several weeks of age,^11^^,^^12^ suggesting symptomatic animals may be amenable to gene-replacement therapy that aims to reconstitute ASPA in its natural lineage compartment. Although we have generated preliminary data supporting this in neonatal animals,^4^ age can have a profound impact on the tropism of individual vector systems.^13^ The current study was undertaken to assess the ability of AAV/Olig001 to target oligodendrocytes in symptomatic animals that model Canavan disease at a stage of the phenotype representative of the clinical condition at the time of diagnosis. Unbiased stereology was used to assess effects of dose and route of administration (ROA) on vector biodistribution and tropism of an AAV/Olig001-GFP reporter vector, with direct pairwise comparisons undertaken to define the optimal combination of dose and ROA for AAV/Olig001 application to Canavan disease. This analysis identified an optimal combination of dose and ROA that was employed to deliver functional AAV/Olig001-ASPA to symptomatic animals and the resulting effects on phenotype assessed relative to treatment with currently available AAV9 technology with documented astrogliotropism and efficacy in preclinical models of Canavan disease.^14^ Identical self-complimentary *aspa* expression cassettes were packaged in both AAV serotypes, with the viral capsid being the sole point of difference. Quantitative metrics of pathologically elevated NAA, rotarod performance, and vacuolation in the brain were used for an objective head-to-head comparison of AAV/Olig001 and AAV9.

## Results

### AAV/Olig001 biodistribution by ROA

**Figure 1:**
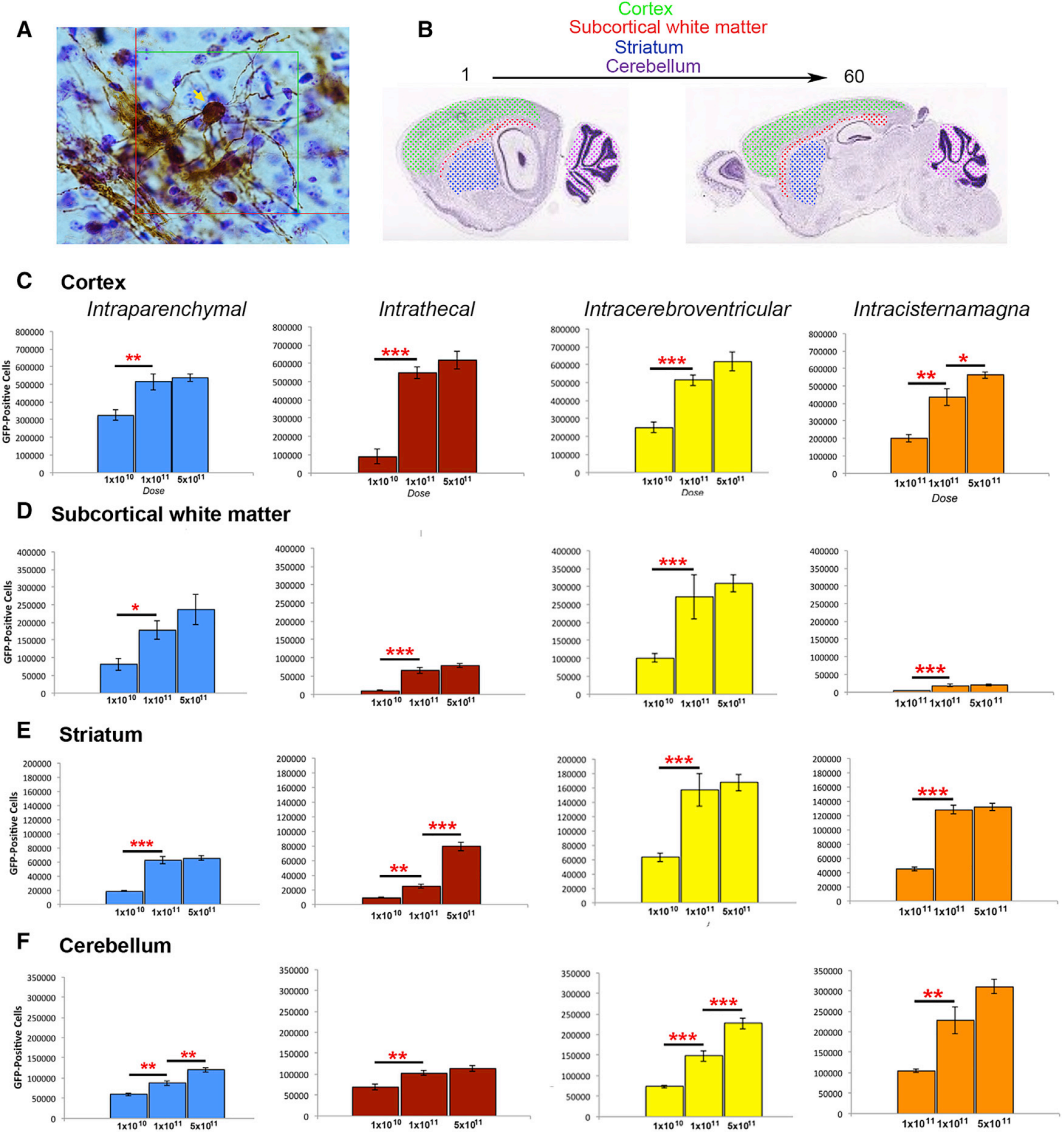
Stereological estimates of AAV/Olig001-GFP transduction via the four indicated ROAs (A) Representative image of a GFP-positive cell at 100× magnification with an optical dissector counting frame. Positive soma (arrow) were counted throughout each region of interest (ROI). (B) Cortical, subcortical white matter (SCWM), striatal, and cerebellar ROIs were sampled (respective color codes highlight sampled regions) for GFP-positive soma in a total of 60 serial sections per brain, with a sampling interval (k) of 4. (C–F) Stereological estimates of GFP-positive cells in the cortex (C), SCWM (D), striatum (E), and cerebellum (F) of animals transduced by the indicated ROAs and at the indicated doses. Significant dose-dependent differences within each ROA cohort are denoted by red asterisks (∗p ≤ 0.05; ∗∗p ≤ 0.01; ∗∗∗p ≤ 0.001). For each dose cohort, n = 5 animals, with mean number ± SEM presented.

**Figure 2:**
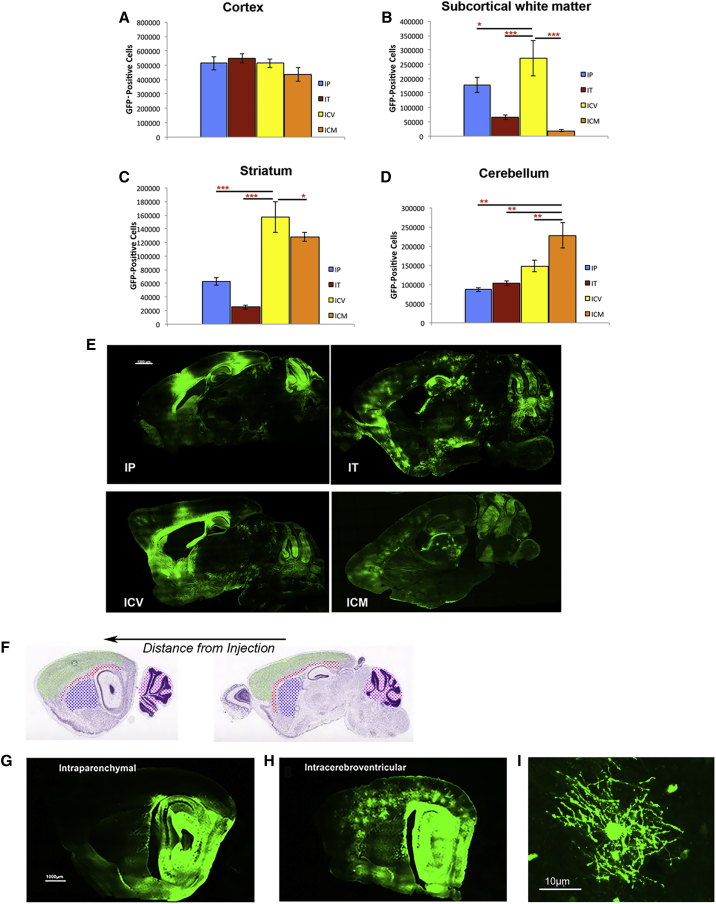
Comparison of effect of ROA on transduction by AAV/Olig001-GFP at 1 × 10^11^ dose in each of the ROIs (A–D) Cortex (A), SCWM (B), striatum (C), and cerebellum (D). Mean ± SEM of n = 5 shown. Significant differences between ROA in each ROI are indicated by red asterisks. (E) Native GFP fluorescence in sagittal section brains transduced with 1 × 10^11^ AAV/Olig001-GFP vector genomes (vgs) via intraparenchymal (IP), intrathecal (IT), intracerebroventricular (i.c.v.), and intracisternamagna (ICM) routes. The IP and i.c.v. routes gave comparably high numbers of GFP-positive cells, but the i.c.v. ROA resulted in much greater spread from injection sites. (F–H) Schematic showing anatomy of sections distal to injection sites and examples of GFP transduction in distal sections (F) from IP (G) and i.c.v. (H) brains. (I) High magnification image of a single cortical GFP-positive cell from the early i.c.v. section shown in (H) highlighting characteristic oligodendrocyte multiprocess-bearing morphology.

6-week-old nur7 animals were given three doses of AAV/Olig001-GFP (1 × 10^10^, 1 × 10^11^, and 1 × 10^10^ total vector genomes [vgs]), each delivered in a volume of 5 μL via one of 4 distinct ROAs: intraparenchymal (IP), intrathecal (IT), intracerebroventricular (i.c.v.), or intracisternamagna (ICM). IP administrations required 5 individual injections of 1 μL each targeting subcortical white matter (SCWM) in both hemispheres and the cerebellum, i.c.v. administration required two 2.5 μL injections to the ventricles of each hemisphere, whereas IT and ICM administration both required a single injection of 5 μL. Transduced animals were sacrificed 2 weeks post-transduction (8 weeks of age) and brains processed for GFP immunohistochemistry and GFP-positive soma (Figure 1A) in the cortex, SCWM, striatum, and cerebellum scored by unbiased stereology (Figure 1B) using the optical fractionator to provide absolute estimates of transduced cells in each region of interest (ROI) (Figures 1C−1F). For all ROAs, an increase in transduced cells was evident when dose was increased from 1 × 10^10^ to 1 × 10^11^ doses in all ROAs, but the highest 5 × 10^11^ dose resulted in only modest additional increases for all ROAs, indicating a ceiling effect in most ROIs. Cortical transduction was comparable across all 4 ROAs, but marked differences in transduction of cells within SCWM of the corpus callosum and external capsule were evident with both the IT and ICM ROA being relatively poor in in this ROI. Striatal transduction was high in i.c.v. and IT groups, whereas ICM administration gave the highest levels of cerebellar transduction. A direct comparison of all four ROAs at the 1 × 10^11^ dose in each ROI revealed clear differences in absolute numbers of transduced cells in all four ROIs (Figure 2). Numbers of GFP-positive cells in the cortex of brains transduced with 1 × 10^11^ vg averaged 440,000−500,000 positive cell soma for all ROAs (Figure 2A). Numbers of positive cells in SCWM tracts differed significantly between ROA cohorts (Figure 2B). i.c.v.- and IP-transduced brains gave the highest and second-highest numbers of transduced white matter tract cells, respectively, with the average 2.7 × 10^5^ positive cells in i.c.v. ROA being significantly greater than the average 1.8 × 10^5^ positive cells present in IP brains (p = 0.041). IT and ICM ROA were both disappointingly inefficient at transducing SCWM cells. The average 1.9 × 10^4^ GFP-positive SCWM cells in the ICM group were 14-fold less than i.c.v. brains (p = 0.000083) and in the IT group 4-fold less (p = 0.0001). The i.c.v. ROA efficiently transduced cells in the striatum (Figure 2C), resulting in higher numbers of striatal GFP-positive soma than any other ROA (i.c.v. versus IP, p = 3.68 × 10^−5^; i.c.v. versus IT, p = 1.61 × 10^−5^; i.c.v. versus ICM, p = 0.043). The efficiency of transduction of the cerebellum was comparable across all IP, IT, and i.c.v. ROAs (Figure 2D), but ICM administration resulted in the highest numbers of transduced cerebellar cells (ICM versus i.c.v., p = 0.045). Although IP and i.c.v. ROA brains were comparable in absolute numbers of cells transduced by AAV/Olig001-GFP in specific regions, the bulk of positive cell counts in IP brains was found in sections immediately adjacent to injection sites, whereas positive cells in i.c.v. brains were found relatively evenly distributed throughout all sections sampled, including those sections (Figures 2F and 2G). Intrasample variance (coefficient of error [CE]) for IP brains was ∼12% of total variance, whereas that for i.c.v. brains was ∼3%, meaning GFP-positive cells were more evenly distributed across all sections sampled in i.c.v. brains. In IP brains, numbers of positive cells in individual sections became fewer the further laterally from injection sites the sampled section was (Figure 2F). The net result of this difference was a greater spread of vector in i.c.v. ROA brains relative to IP brains, particularly in the cortex and SCWM (Figures 2G and 2H). The i.c.v. ROA resulted in the highest number of total GFP-positive cells (sum of all ROI counts in individual brains), which were 1.3-fold more than the next-ranked ROA, IP (p = 0.0067). Total numbers in i.c.v. brains were significantly increased over all ROAs, including ICM (p = 0.0027) and IT (p = 0.0003). Numbers of cells in IP ROA brains were not significantly increased over either ICM (p = 0.730) or IT numbers (p = 0.165), marking the i.c.v. ROA clearly superior in total cells transduced. Approximately 75% of the difference in overall GFP-positive cell numbers between i.c.v. and IP cohorts (∼262,891) was accounted for by SCWM (35%) and striatal (36%) ROIs, which manifest >80% oligotropism in both ROA cohorts. This means that i.c.v. brains contained somewhere in the region of at least 210,000 more transduced oligodendrocytes than IP brains. If this analysis is restricted to within SCWM, an ROI presenting >90% oligotropism by all ROAs, then at least 83,000 more transduced oligodendrocytes per brain are to be expected when administering AAV/Olig001 via the i.c.v. ROA. When assessed against the ROA cohort presenting the poorest levels of GFP transgene expression, the ICM cohort, i.c.v. administration results in an increase in AAV/Olig001-transduced oligodendrocytes of over 200,000 cells per brain. Total GFP-positive cells for combined ROIs using each of the 4 ROA cohorts at the 1 × 10^11^ dose are summarized in Table 1.

**Table 1:** Group mean GFP-positive cells in brains subject to indicated ROA (n = 5), with standard deviations (SDs) given in parentheses

ROA	Mean total GFP-positive cells (SD)
i.c.v.	1,104,256.4 (106,816.96)
IP	841,365.6 (121,722.7)
ICM	815,486.9 (106,979.7)
IT	742,143.1 (79,496.5)

**Figure 3:**
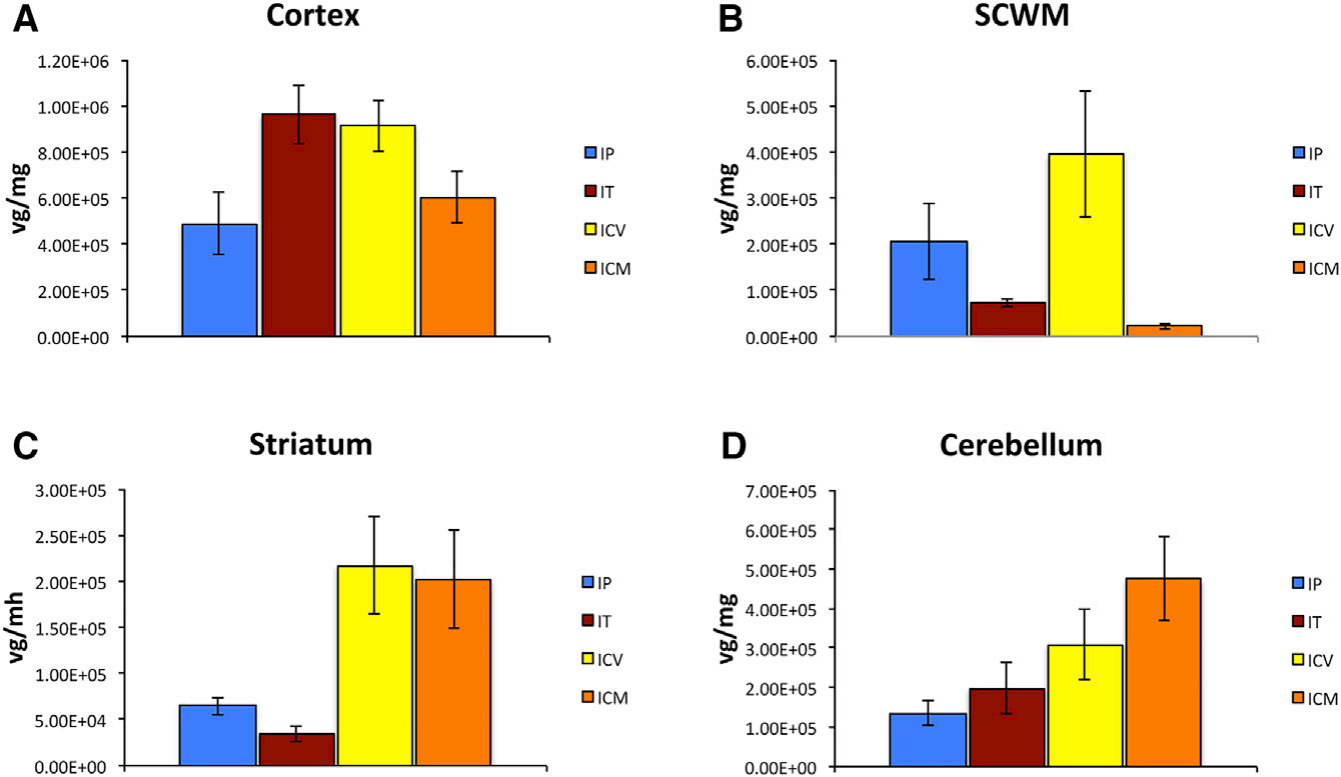
Analysis of vg copy number in indicated regions for each ROA cohort receiving 1 × 10^11^ total vgs Mean vg copy number per group presented as vg per milligram wet tissue weight (n = 4). Tissue was dissected from each ROA in 22-week-old brains after transduction at 6 weeks of age with AAV/Olig001-GFP. DNA was isolated and real-time PCR performed using a custom-designed TaqMan probe/primer set specific for the bovine growth hormone (BGH) polyadenylation sequence in the transgene expression cassette. (A–D) vg number per milligram of wet tissue weight is presented for the cortex (A), SCWM (B), striatum (C), and cerebellum (D). The mean ± SEM is presented for each indicated ROA.

To explore the relationship between number of cells transduced and the efficiency of transduction, each individual ROI (cortex, SCWM, striatum, and cerebellum) in AAV/Olig001-GFP brains receiving a 1 × 10^11^ dose was dissected and processed for analysis of vg copies by real-time PCR using a custom TaqMan probe/primer set targeted to the bovine growth hormone polyadenylation (BGH poly(A)) sequence of the genome (Figure 3). Differences in copies of vg detected in each ROI generally followed the pattern of stereological GFP-positive estimates with the exception of the cortex. In the cortex, nonsignificant differences in total GFP-positive cells via all ROAs (Figure 2A) contrasted with significant differences in vg copy number between some ROAs. Cortical GFP-positive cell numbers in IT brains were not significantly different from the same in IP brains, but vg per milligram of cortical tissue in IT brains was significantly greater than vg per milligram of cortical tissue in IP brains (p = 0.042), suggesting superior cortical diffusion via the IP ROA, despite resulting in approximately one-half the number of available vgs. Similarly, cortical GFP-positive cell numbers in i.c.v. brains were identical to that in IP brains, but vg copy number was significantly higher (p = 0.05), also pointing to superior diffusion of vector via the IP ROA. In all other ROIs, relative numbers of GFP-positive cells in all ROA cohorts followed closely relative vg copy number.

### Tropism of AAV/Olig001-GFP compared by ROA

**Figure 4:**
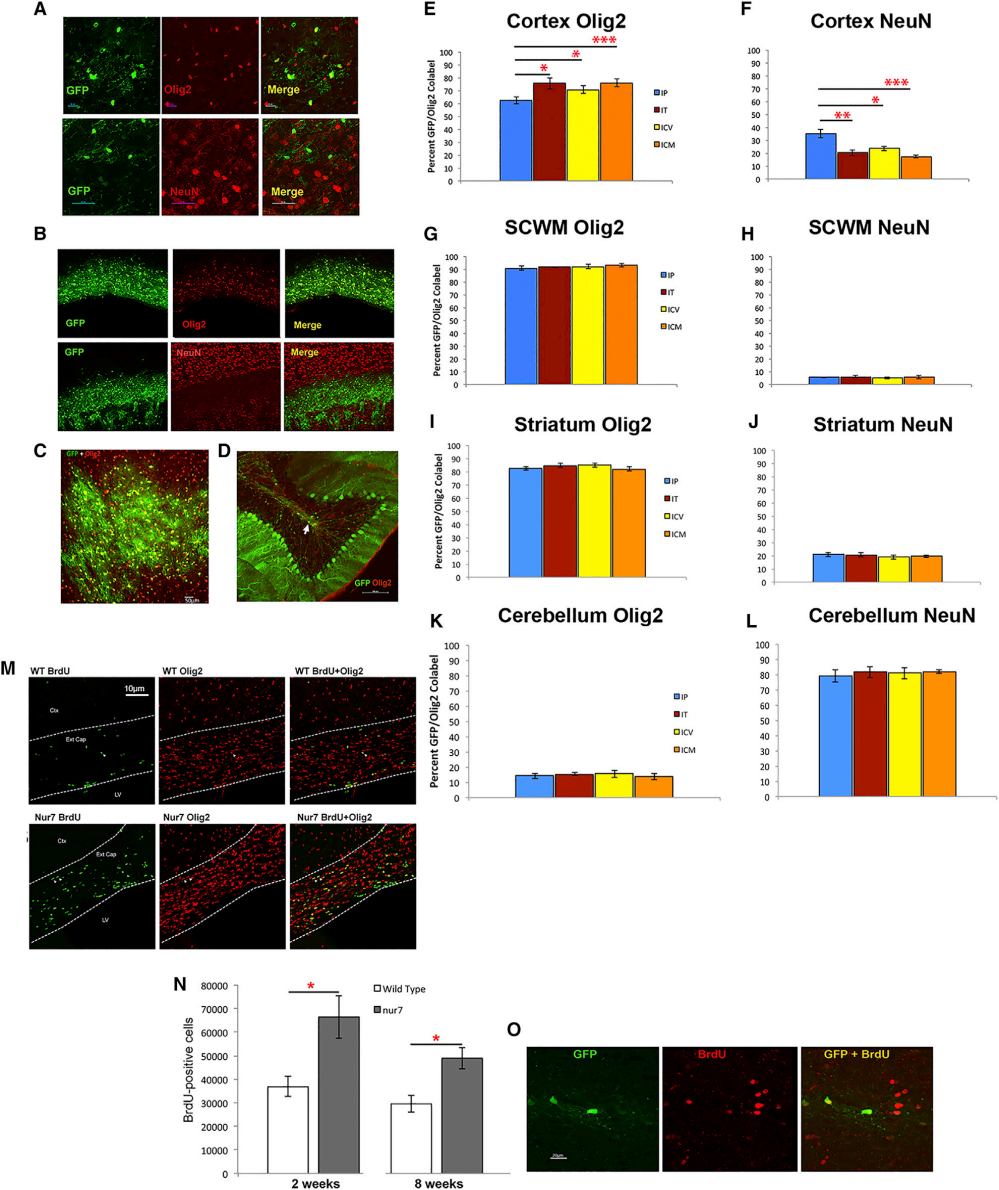
Effect of ROA on tropism of AAV/Olig001-GFP delivered at a 1 × 10^11^ dose via the indicated ROA Dual channel stacks of GFP/Olig2-positive and GFP/NeuN-positive cells were collected by systematic sampling and colabeled entities scored using the optical fractionator in the cortex, SCWM, striatum, and cerebellum. (A and B) Example of differing degree of Olig2 and NeuN colabeling with native GFP in the cortex (A) and SCWM (B). (C and M) Striatal GFP colabeling was predominantly Olig2 in the striatum (C), but Olig2 colabeling in the cerebellum (D) was relatively rare . (E–L) Stereological estimates of GFP-Olig2 colabeling in the cortex (E), SCWM (G), striatum (I), and cerebellum (K) alongside GFP-NeuN colabeling in these regions in adjacent sections (F, H, J, and L, respectively). Significant differences in GFP colabeling are denoted by red asterisks. For each dose cohort, n = 5, with mean number ± SEM presented. (N) Stereological estimates of BrDU-positive cells within white matter tracts of wild type and nur7 mice at indicated ages. Mean + SEM presented, n=5 per genotype at each age. (O) representative co-labeling of GFP and BrdU (red) in SCWM of the nur7 brain 2 weeks after transduction with AAV/Olig001-GFP at 6 weeks of age.

The main point of difference of AAV/Olig001 and currently available AAV capsid serotypes is the inherent oligotropism of the former. For clinical application to white matter disease, AAV/Olig001 vectors must be shown capable of faithfully replicating this tropism in preclinical models of specific diseases. Assumption of intact oligotropism would be unwise given significant variation can result from variables such as age of intervention,^13^^,^^15^ and although our previous work has documented the oligotropic potential of AAV/Olig001 in neonatal nur7 mice,^4^ translation of this tropic potential to older, symptomatic animals remains untested. To this end, all four ROAs employed to deliver a 1 × 10^11^ dose of AAV/Olig001-GFP in the current study were assessed for impact on vector tropism in 6-week-old animals (Figure 4). The cortex, SCWM, striatum, and cerebellum of the same brains used for the generation of absolute numbers of GFP-positive cells were analyzed for colabeling of native GFP fluorescence with lineage-specific antigens. On the whole, all four ROAs generated comparable results, with inherent AAV/Olig001 oligotropism largely intact. Nonoligodendrocyte transgene expression was attributable in the main to neuronal expression, with very few astrocytes observed expressing GFP in either of the 4 ROA cohorts (<5%). In the cortex, 62.3% of all GFP-positive cells colabeled with Olig2 and 35.1% with NeuN in IP brains. In IT ROA brains, 75.5% of cortical GFP-positive cells colabeled with Olig2 and 20.2% with NeuN. i.c.v. ROA brains presented with 70.8% oligotropism and 23.6% neurotropism in the cortex, whereas the cortex of ICM brains manifest 76% GFP colabeling with Olig2 and 17.4% with NeuN. The difference in oligotropism manifest among the 4 different ROAs was small, but the IP ROA did present with a significant increase in NeuN colabeling (p = 0.0043 versus IT; p = 0.0119 versus i.c.v.; p = 0.00059 versus ICM) that coincided with slight but significant reductions in Olig2-colabeling relative to the other 3 ROAs (p = 0.026 versus IT; p = 0.048 versus i.c.v.; p = 0.0085 versus ICM), suggesting the IP ROA promotes small increases in neurotropism at the expense of oligotropism. Most of the GFP-NeuN colabeling in IP ROA brains was clustered around injection sites, indicating saturating quantities of AAV/Olog001-GFP immediately adjacent to the site of injection. Tropism in SCWM for each ROA cohort was identical for all 4 ROAs. All 4 of IP, IT, i.c.v., and ICM ROAs resulted in >90% oligotropism, <6% neurotropism, with no significant differences in percent colabeling with either antigen, indicating a strong preference for oligodendrocytes in white matter-rich regions regardless of ROA. Striatal tropism was also comparable for all 4 ROAs, with >80% of all GFP-positive cells colabeling with Olig2 and <20% with NeuN. The cerebellum presented with starkly contrasting tropism for all 4 ROAs, with a mere 10% of all GFP-positive cells colabeling with Olig2 and over 80% colabeling with NeuN. No significant differences in percent colabel with either antigen were observed between ROA cohorts in the cerebellum. Cerebellar transduction was dominated by large Purkinje neurons in the granule cell layer, with relatively sporadic oligodendrocyte transgene expression in white matter tracts. This is in contrast to the near 100% oligotropism observed in SCWM and the 70%–80% oligotropism observed in comparatively neuron-dense regions such as the cortex and striatum, marking the cerebellum as somewhat refractory to oligodendrocyte-restricted transduction with AAV/Olig001.

The adult mammalian CNS is known to harbor significant numbers of oligodendrocyte precursor cells in white matter,^16^ and we have previously shown evidence of attempted remyelination in juvenile nur7 in the form of an increased turnover of immature oligodendrocytes.^12^ In addition, SCWM in Canavan patients presents with severe abnormalities, such as a swollen aspect, and progressive fractional anisotropic abnormalities, as documented by diffusion tensor imaging (DTI).^7^ Given that white matter has a significant capacity for remyelination, even in the adult brain, the persistence of a resident population of immature oligodendrocytes in adult nur7 white matter must be considered an ideal target for an oligotropic gene-delivery vector. In order to assess relative numbers of proliferating oligodendrocyte progenitors/immature oligodendrocytes, both nur7 and wild-type mice were given systemic bromodeoxyuridine (BrdU) twice a day for 2 days and sacrificed on the third day to process for BrdU/Olig2 colabeling (Figures 4N and 4O). BrdU administration was initiated in both 2- and 8-week-old cohorts to quantify the possible persistence of proliferating oligodendrocytes in young and adult brains. Counts of BrdU-positive cells in the corpus callosum and external capsule of genotype cohorts at each age revealed a significant 1.8-fold increase in BrdU-positive cells in 2-week-old nur7 brains relative to wild type (p = 0.029) and a 1.6-fold increase in nur7 brains at 8 weeks (p = 0.034). The vast majority of BrdU cells in nur7 white matter, at both ages, colabeled with Olig2, indicating the persistence of proliferating progenitor/immature oligodendrocytes in white matter of adult symptomatic nur7 mice. A subset of three 6-week-old nur7 mice was given systemic BrdU for 2 days prior to transduction with 1 × 10^11^ vg of AAV/Olig001-GFP, and these animals were sacrificed 2 weeks post-transduction for evidence of transduction of proliferating cells in white matter tracts (Figure 4N). Numerous BrdU/GFP colabeled cells were observed in white matter tracts of these animals, indicating transduction of resident progenitor/immature cells.

In order to assess oligotropism of AAV/Olig001-GFP in the nur7 brain in the context of percentage of resident oligodendrocytes transduced, 8-week-old naive nur7 brains (n = 5) were analyzed for resident Olig2-positive cells using sampling parameters identical to those employed for the analysis of numbers of GFP-positive cells in AAV/Olig001-GFP-transduced brains to calculate endogenous Olig2 content in each ROI at the time of analysis (Table 2). These estimates of Olig2 cell number are absolute and independent of volume density, meaning bias in possible differences in tissue volume produced by GFP staining in AAV/Olig001-GFP cohorts is avoided.

**Table 2:** Stereological estimates of Olig2-positive cells in the indicated ROI in 8-week-old naive nur7 mice

Resident cortical Olig2 population	Resident SCWM Olig2 population	Resident striatal Olig2 population	Resident cerebellar Olig2 population
Mean: 183,095	mean: 913,428	mean: 136,252	mean: 344,651
SD: 22,963	SD: 104,177	SD: 136,308	SD: 55,144

The mean and SD are given (n = 5). Mean rounded to the nearest whole number.

Numbers of resident oligodendrocytes thus calculated were then used to determine the percentage of these cells transduced by AAV/Olig001-GFP via each ROA. The percent oligotropism determined by colabeling analysis (Figures 4E−4L) was then calculated as a proportion of total GFP-positive cells in each region at each dose (Figure 1), and this proportion was used to calculate the percent of total resident Olig2 cells transduced at each ROI (Table 3). The percent resident oligodendrocytes transduced was dose responsive for each ROA but with the increase from 1 × 10^11^ to 5 × 10^11^ dose being very modest. The lowest percentage transduction was seen in the cerebellum, with 5% for IP brains to 18% for ICM brains. In SCWM, only 2% of resident oligodendrocytes was transduced in the ICM brain compared with 31% in i.c.v. brains. Likewise, the i.c.v. ROA resulted in transduction of 10% of resident oligodendrocytes, whereas IP administration resulted in transduction of only 4% of the resident oligodendrocyte population. Cortical transduction was spread from 18% to 25%, with the ICM ROA resulting in the highest levels of oligodendrocyte transduction. Overall, the i.c.v. ROA appeared to be superior for consistently high levels of transduction of resident oligodendrocytes.

**Table 3:** Percentage of resident oligodendrocytes transduced in each ROI by AAV/Olig001-GFP at the indicated doses for each ROA

	ROA	Dose	Total GFP	GFP/Olig2	Olig2 (%)
Cortex	IP	1 × 10^10^	325,472.3	20,276,924.3	10.90
		1 × 10^11^	513,476.7	31,989,598.4	17.30
		5 × 10^11^	537,015.8	33,456,084.3	18.10
	IT	1 × 10^10^	90,599.6	68,402.7	3.70
		1 × 10^11^	54,905.5	414,536.5	22.40
		5 × 10^11^	617,330.5	466,084.5	25.20
	i.c.v.	1 × 10^10^	251,262.9	177,894.1	9.60
		1 × 10^11^	513,815.2	363,781.2	19.60
		5 × 10^11^	618,182.5	437,673.2	23.60
	ICM	1 × 10^10^	200,934.9	152,710.5	8.24
		1 × 10^11^	435,536.5	331,007.7	17.90
		5 × 10^11^	562,036.5	427,147.7	23.10
SCWM	IP	1 × 10^10^	81,504.2	79,205.8	8.70
		1 × 10^11^	178,362.4	173,332.6	19.00
		5 × 10^11^	236,390.6	229,724.4	25.10
	IT	1 × 10^10^	10,340.8	9,482.5	1.04
		1 × 10^11^	64,970.3	59,577.8	6.50
		5 × 10^11^	79,443.9	72,850.1	8.00
	i.c.v.	1 × 10^10^	101,562.9	93,437.9	10.20
		1 × 10^11^	271,274.4	249,572.4	27.30
		5 × 10^11^	308,557.9	283,873.3	31.10
	ICM	1 × 10^10^	3,675.9	3,425.9	0.38
		1 × 10^11^	18,996.3	17,704.6	1.94
		5 × 10^11^	19,870.5	18,519.3	2.03
Striatum	IP	1 × 10^10^	18,679.4	15,386.2	1.13
		1 × 10^11^	62,706.5	51,651.3	3.79
		5 × 10^11^	65,202.7	53,707.5	3.94
	IT	1 × 10^10^	9,436.5	8,002.2	0.59
		1 × 10^11^	25,154.6	21,331.1	1.57
		5 × 10^11^	79,443.9	67,368.4	4.94
	i.c.v.	1 × 10^10^	63,111.8	536,450.3	3.94
		1 × 10^11^	157,202.6	133,622.2	9.81
		5 × 10^11^	167,171.9	142,096.1	10.40
	ICM	1 × 10^10^	44,990.8	36,937.4	2.71
		1 × 10^11^	128,160.3	1,052,219.6	7.72
		5 × 10^11^	132,020.1	108,388.5	7.95
Cerebellum	IP	1 × 10^10^	58,903.6	8,317.2	2.41
		1 × 10^11^	86,820.1	1,225.9	3.56
		5 × 10^11^	119,628.9	16,891.6	4.90
	IT	1 × 10^10^	68,367.3	10,555.9	3.06
		1 × 10^11^	102,963.2	15,897.5	4.61
		5 × 10^11^	113,843.9	17,577.5	5.10
	i.c.v	1 × 10^10^	73,111.8	11,405.4	3.31
		1 × 10^11^	148,148.6	23,111.2	6.71
		5 × 10^11^	227,171.9	35,438.8	10.30
	ICM	1 × 10^10^	105,220.1	14,520.4	4.21
		1 × 10^11^	228,282.3	3,150.3	9.14
		5 × 10^11^	310,289.4	42,819.9	12.40

Total GFP-positive cells scored at each dose for each ROA (Figure 1) were converted to number of Olig2/GFP-colabeled cells based on percent tropism, and this figure was used to calculate the percentage of total resident cells in each ROI (Table 2) transduced.

### Head-to-head comparison of phenotypic rescue in AAV/Olig001-ASPA- and AAV9-ASPA-transduced nur7 mice

**Figure 5:**
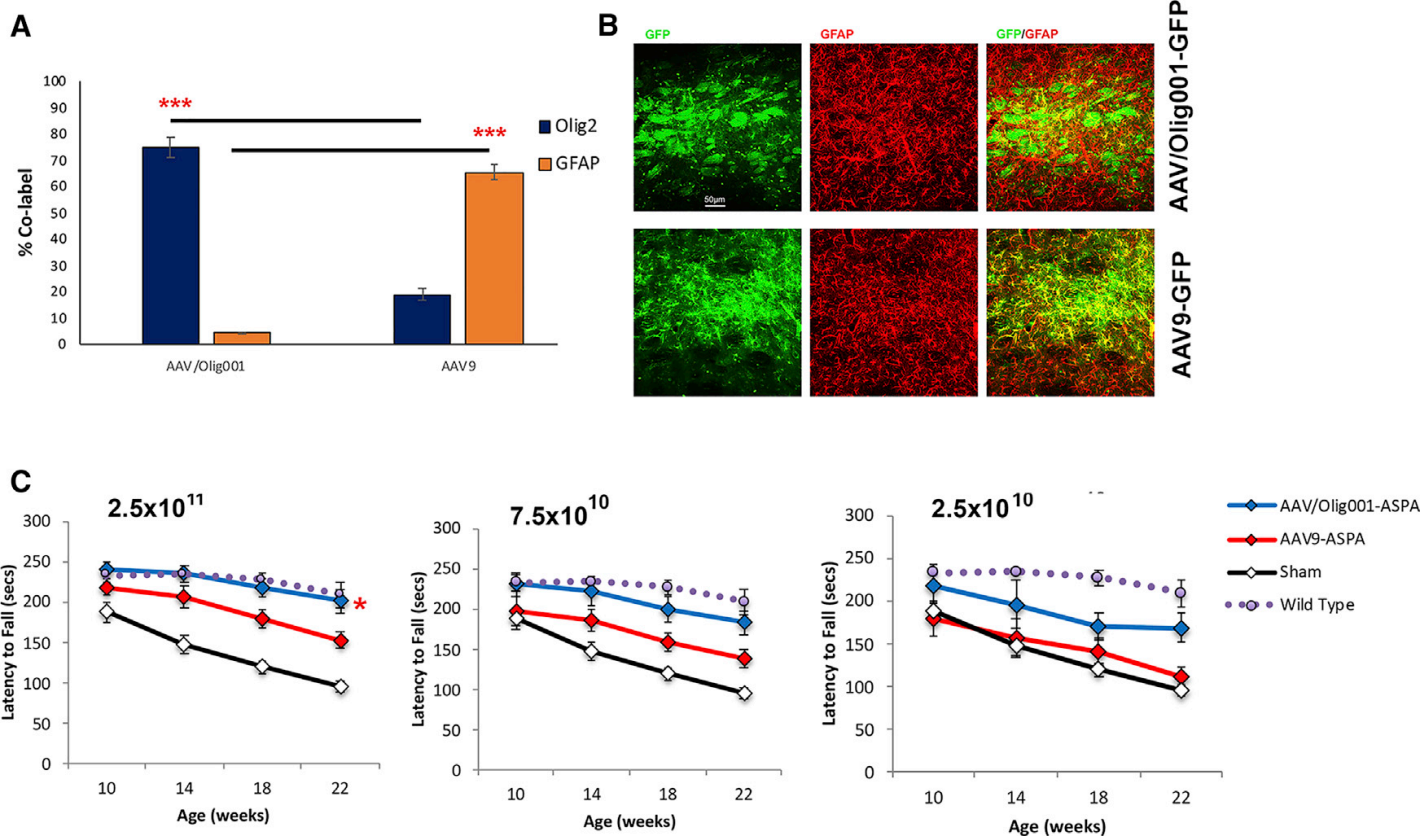
Striatal injection of 5 × 10^10^ vg of either AAV/Olig001-GFP or AAV9-GFP results in contrasting glial tropism (A) Mean percent colabeling with either Olig2 (blue) or GFAP (orange), ± SEM presented (n = 4). (B) Representative images of GFP-GFAP colabeling in AAV/Olig001-GFP-transduced brains (upper 3 panels) and AAV9-GFP-transduced brains (lower 3 panels), showing higher levels of GFAP colabeling in AAV9 brains. (C) Animals treated at the indicated doses with AAV/Olig001-ASPA (blue) or AAV9-ASPA (red) and assessed for rotarod latency to fall at 10, 14, 18, and 22 weeks of age. Sham nur7 (purple) and naive wild-type controls (black) are included. Mean ± SEM for each group presented (n = 12). Red asterisk indicates significant improvement in AAV/Olig001-ASPA-treated animals, as determined by repeated-measures ANOVA.

Biodistribution and tropism analysis of AAV/Olig001-GFP points to a clear advantage for the i.c.v. ROA. A codon-optimized human *aspa* coding sequence (CDS) was packaged into self-complimentary AAV/Olig001 for i.c.v. delivery to 6-week-old nur7 mice and also packaged into a self-complimentary AAV9 vector containing identical transcriptional regulatory elements for a head-to-head comparison of phenotypic rescue. AAV9 has been recently reported to rescue motor function in another Canavan mouse model by way of astroglial transduction, thus providing a significant point of difference with regard to choice of serotype.^14^ In order to confirm tropism as a point of difference between AAV/Olig001 and AAV9, the same GFP expression cassette used for AAV/Olig001 biodistribution studies was packaged into AAV9, and both AAV/Olig001-GFP and AAV9-GFP were assessed for tropism when injected in the striatum of 6-week-old nur7 mice (Figure 5A). Although AAV/Olig001-GFP promoted oligodendroglial expression of GFP in 75% of all transgene-positive cells, AAV9-GFP promoted oligodendroglial GFP expression in only 19% of all transgene-positive cells, a nearly 4-fold difference (p = 1.64 × 10^−5^). Only 4% of all GFP-positive cells in AAV/Olig001-GFP brains colabeled with glial fibrillary acidic protein (GFAP), compared with 65% in AAV9-GFP brains (p = 8.88 × 10^−7^), thereby distinguishing AAV9 as predominantly astrogliotropic. Codon-optimized human *aspa* CDS packaged into identical self-complimentary AAV expression cassettes in both AAV/Olig001 and AAV9 (AAV/Olig001-ASPA and AAV9-ASPA) was administered at three doses (2.5 × 10^11^, 7.5 × 10^10^, and 2.5 × 10^10^) to 6-week-old nur7 mice via the i.c.v. ROA for head-to-head comparison of efficacy. The maximum dose was determined by the upper limit of vg that could be delivered in a volume of 5 μL, with two successive ∼3-fold reductions. Motor function was assessed by latency to fall from an accelerating rotarod at 10, 14, 18, and 22 weeks of age to compare dose-responsive effects of each vector system. Age-matched sham-treated nur7 mutants and age-matched naive wild-type animals provided negative and reference controls. At the highest dose administered, both AAV/Olig001-ASPA and AAV9-ASPA promoted significant improvements over sham nur7 control latencies to fall at all four time points tested by unpaired comparison of means (Table 4). However, AAV/Olig001-ASPA-treated animals were indistinguishable from wild-type reference controls at all ages, whereas AAV9-ASPA-treated animals presented significantly reduced latency to fall relative to wild-type at 18 and 22 weeks of age, indicating a lower threshold effect. Analysis of within-subject means across the entire 5.5 month in life phase by repeated-measures ANOVA revealed AAV/Olig001-ASPA motor function to be significantly improved relative to sham controls at the highest dose (p = 0.028), whereas motor function in AAV9-ASPA animals receiving an equivalent dose was not (p = 0.551). At the mid-dose (7.5 × 10^10^), both vectors promoted consistently improved latency to fall at all ages, but effects of AAV/Olig001-ASPA treatment appeared more significant. At the two latter time points (18 and 22 weeks), when sham nur7 motor function was severely compromised, AAV/Olig001-ASPA mean latencies to fall were significantly greater than AAV9-ASPA animals (p = 0.00018 and p = 0.027, respectively). At the lowest dose administered (2.5 × 10^10^), only AAV/Olig001-ASPA treatment resulted in significantly increased latency to fall and only at the 18- and 22-week time points, when motor dysfunction in controls was at its most pronounced.

**Table 4:** Mean latencies to fall from an accelerating rotarod in groups of animals of the indicated treatment and dose cohorts (n = −12/group)

2.5 × 10^11^	Age: weeks			
AAV/Olig001	10	14	18	22
Mean	239.8	234.9	217.8	201.6
SD	36.7	32.3	41.1	50.2
p value versus sham	0.0039	6.59 × 10^−7^	6.06 × 10^−6^	1.23 × 10^−6^

**AAV9**				

Mean	216.7	206.2	178.7	153
SD	26	48.1	11.4	36.8
p value versus sham	0.055	0.0033	0.0003	0.00017
7.5 × 10^10^				

**AAV/Olig001**				

Mean	230.3	222.7	199.8	183.3
SD	52.1	64.7	55.7	50.4
p value versus sham	0.039	0.0027	0.00018	1.87 × 10^−5^

**AAV9**				

Mean	197.1	185.4	159.7	139.3
SD	62	46.4	40.5	39.8
p value versus sham	0.676	0.047	0.009	0.0037
2.5 × 1010				

**AAV/Olig001**				

Mean	218.25	195.9	171.1	169.1
SD	68.6	97.6	55.7	55.8
p value versus sham	0.205	0.133	0.0085	0.0004

**AAV9**				

Mean	179	157.3	140.8	110.7
SD	68.7	78	57.6	45.1
p value versus sham	0.704	0.727	0.257	0.33
Controls				

**Sham**				

Mean	187.9	148.4	119.4	96.1
SD	41.8	39.5	27.2	23.5
p value versus wild type	0.205	0.133	0.0085	0.0004

**Wild type**				

Mean	232.9	234.1	227.5	208.8
SD	34.5	37.8	31	55.5
p value versus sham	0.0088	1.87 × 10^−5^	6.85 × 10^−9^	1.61 × 10^−6^

Mean and SD are given, as well as pairwise comparisons (p value) of indicated groups.

**Figure 6:**
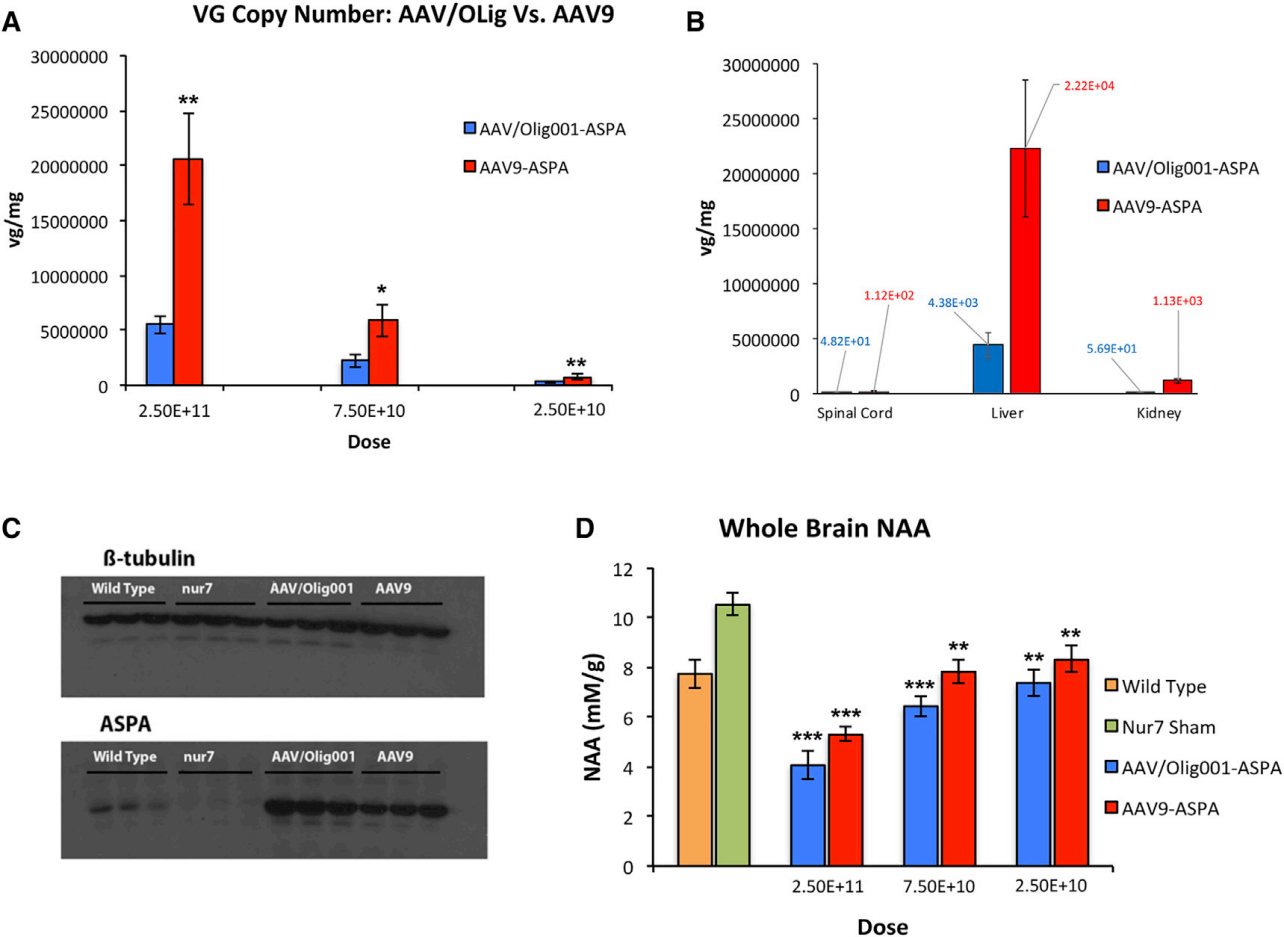
AAV/Olig001 and AAV9 Vector genome biodistribution and function (A) vg copy number in whole brains of 22-week-old animals treated with the indicated dose of either AAV/Olig001-ASPA (blue) or AAV9-ASPA (red). vg expressed as copy number per milligram of wet tissue weight (vg/mg), with group mean ± SEM presented (n = 6). Asterisks denote significant differences in vg copy number at the indicated dose. (B) vg copies per mg of tissue from the spinal cord, liver, and kidney of AAV/Olig001-ASPA and AAV9-ASPA 22-week-old animals administered via the ROA at the 2.5 × 10^11^ dose. Group means for each tissue are indicated in blue and red script (n = 6). (C) Reconstitution of ASPA protein in both AAV/Olig001-ASPA- and AAV9-ASPA-treated 22-week-old nur7 brains. Sham nur7 negative controls show the absence of detectable protein. (D) Whole-brain NAA in indicated treated and control groups at 22 weeks of age. The decrease of the dose of both AAV/Olig001-ASPA (blue) and AAV9-ASPA (red) results in a corresponding increase in NAA. Age-matched wild-type naive brains (orange) and sham nur7 brains (green) are provided to highlight chronically elevated NAA in nur7 brains. Mean ± SEM for each group provided (n = 6).

At the conclusion of the 22-week rotarod time point, animals were sacrificed and brains analyzed for vg copy number, ASPA content, and whole-brain NAA (Figure 6). Transduction with both vectors gave a dose-dependent increase in detectable vgs (Figure 6A), with AAV9-ASPA-transduced brains containing significantly more detectable genomes at each dose. The genome copy number was 2.5- to 3-fold greater in AAV9-ASPA brains at each of 2.5 × 10^11^, 7.5 × 10^10^, and 2.5 × 10^10^ doses (p = 0.052, p = 0.039, p = 0.003, respectively), indicating a greater efficiency of transduction. Off-target organs were assessed for transduction by each vector serotype at the 2.5 × 10^11^ dose (Figure 6B), with detectable genomes in all of the spinal cord, liver, and kidneys. The liver had, by far, the greatest vg burden, with AAV9-ASPA livers having 5-fold times more genome copies per milligram than AAV/Olig001-ASPA livers (p = 0.018), greater than the difference in brain genome copy number. Abundant levels of ASPA protein were detectable in brains transduced by AAV9-ASPA and AAV/Olig001-ASPA, confirming gene replacement (Figure 6C). Transgene functionality was confirmed in brains transduced with both vector serotypes, with each displaying a dose-dependent reduction in whole-brain NAA to levels significantly below sham nur7 controls (Figure 6D). At the highest dose employed, both vectors promoted a highly significant reduction in NAA relative to sham controls, with a 2.6-fold reduction in AAV/Olig001-ASPA brains (p = 5.06 × 10^−6^) and a 2-fold reduction in AAV9-ASPA brains (p = 1.97 × 10^−6^). These reductions in NAA were so pronounced that they were significantly below wild-type levels in both vector-treated nur7 cohorts (AAV/Olig001: p = 0.001; AAV9: p = 0.003). Despite differences in relative transduction efficiency between the two serotypes, AAV/Olig001-ASPA brains manifest the greater reduction in NAA, but this reduction failed to reach statistical significance at any dose. The presence of detectable ASPA protein and evidence of continued NAA catabolism for over 4 months indicate persistent long-term transgene expression.

**Figure 7:**
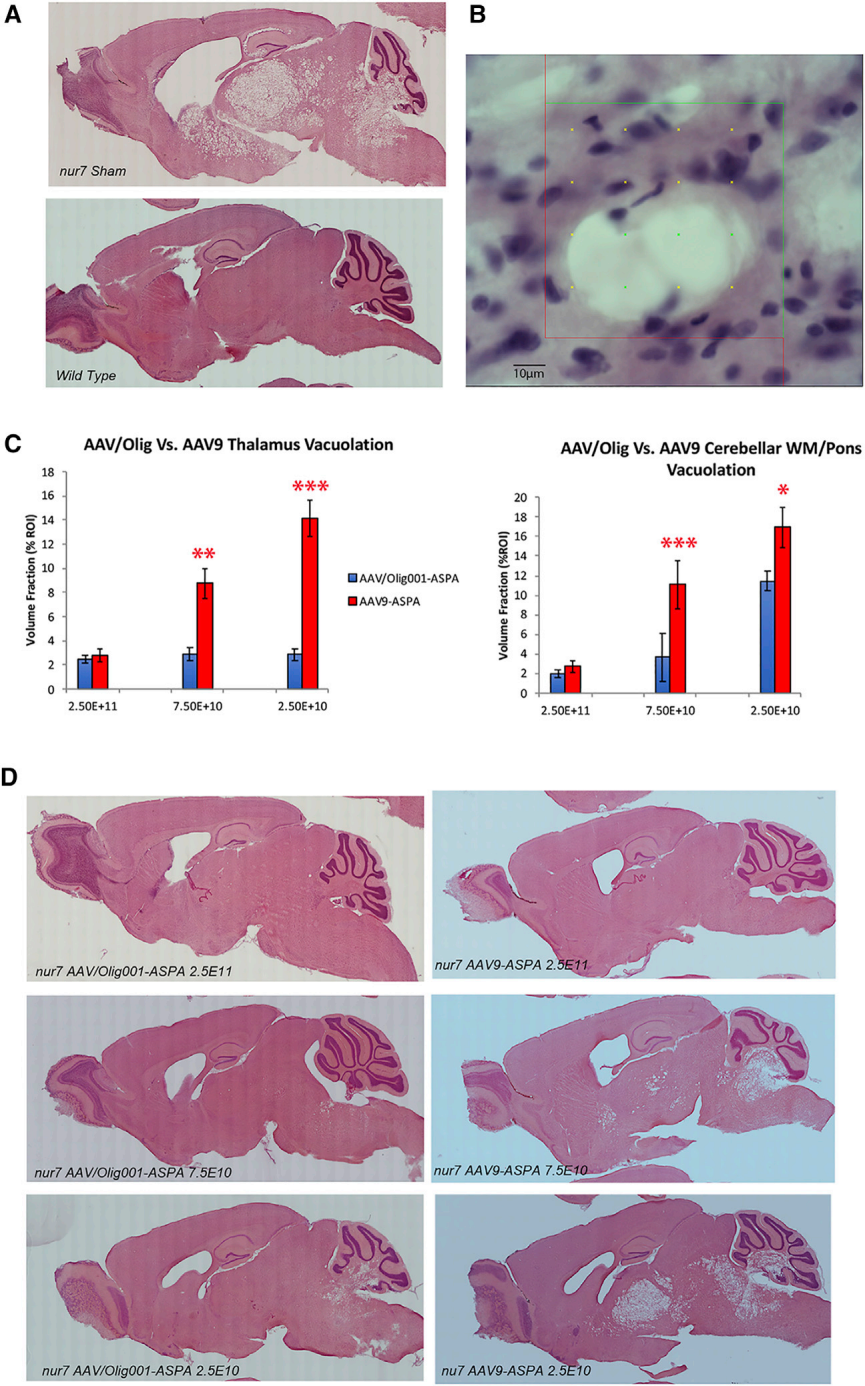
Comparative rescue of vacuolation in AAV/Olig001- and AAV9-treated brains (A) Representative H&E-stained sagittal sections of a 22-week-old nur7 brain (upper) and age-matched wild-type brain (lower) highlighting severe vacuolation in the thalamus, pons, and cerebellar white matter. (B) Vacuole volume fraction in brains was scored using an object volume fraction probe that scored the percentage of ROIs occupied by vacuoles in H&E-stained sections. (C) Relative vacuole volume fraction in AAV/Olig001-ASPA (blue)- and AAV9-ASPA (red)-treated 22-week-old brains with data for both the thalamus and pons/cerebral white matter presented. AAV/Olig001 rescued a greater proportion of each region (i.e., greater reduction in vacuole volume) than did AAV9 at all of the three indicated doses. Mean volume fraction (percent of total ROI sampled) shown ± SEM (n = 6). (D) Representative H&E-stained sections from AAV/Olig001-ASPA-treated (left 3 panels) and AAV9-ASPA-treated (right 3 panels) 22-week-old nur7 mice at the indicated doses, showing progressively more vacuolation in AAV9 brains with decreasing dose.

Gross pathology in 22-week AAV/Olig001-ASPA- and AAV9-ASPA-treated brains was then assessed to gauge the relationship between degree of motor function improvement (Figure 7). Vacuolation is a prominent pathological hallmark of Canavan disease, thought to be related to chronically elevated NAA. Serial sections from treated and control brains were processed for H&E staining to highlight vacuolated regions of the thalamus and pons/cerebellum (Figure 7A). 22-week-old wild-type brains did not have any detectable thalamic vacuolation, whereas 22-week-old nur7 sham brains had 33.33% (standard deviation [SD]: 5.1) of thalamic volume occupied by vacuoles. At the highest dose (2.5 × 10^11^), both AAV/Olig001-ASPA and AAV9-ASPA promoted significant reductions in thalamic vacuole volume fraction. AAV/Olig001-ASPA thalamic vacuolation was reduced 13-fold relative to sham controls (p = 4.76 × 10^−8^) and AAV9-ASPA by 12-fold (p = 6.29 × 10^−8^), indicating similar levels of rescue. However, at successively lower doses, clear differences in the degree of vacuole volume rescue become apparent. At the mid-7.5 × 10^10^ dose, thalamic vacuolation in AAV/Olig001-ASPA brains was reduced 12-fold (p = 6.38 × 10^−8^) and by 4-fold in AAV9-ASPA brains (p = 1.49 × 10^−8^). Although the thalamic vacuole reduction in AAV9-ASPA brains was highly significant, it was also 3-fold less than rescue in AAV/Olig001-ASPA brains at the same dose (p = 0.0014). At the lowest 2.5 × 10^10^ dose, both serotypes promoted significant reductions relative to sham controls, but the reduction in AAV/Olig001-ASPA brains was 5-fold greater than that in AAV9-ASPA brains (p = 2.96 × 10^−5^).

In the pons and white matter of the cerebellum, 22-week-old sham nur7 brains manifest a 26.4% (SD: 7.5) vacuole volume fraction. The dose-dependent degree of rescue by AAV/Olig001-ASPA and AAV9-ASPA was much more aligned than as was the case in the thalamus. At the highest dose, pons/cerebral white matter vacuolation was reduced 13-fold in AAV/Olig001-ASPA brains (p = 1.3 × 10^−5^) and 9-fold in AAV9-ASPA brains (p = 1.87 × 10^−5^), marking the two vectors as virtually indistinguishable. Once again, however, whereas both vectors successfully and significantly reduced pons/cerebral white matter vacuolation at successively lower doses, reductions in AAV/Olig001-ASPA brains were 3-fold and 1.5-fold greater than AAV9-ASPA at 7.5 × 10^10^ and 2.5 × 10^10^, respectively (p = 7.99 × 10^−6^; p = 0.037). Thus, observed differences in the rescue of motor abnormalities between the two vector serotypes appear to translate to differences in the degree of rescue of gross pathology.

## Discussion

The relative inefficiency of oligodendroglial transduction by currently available viral vectors is a significant unmet need for white matter disease gene therapy. The AAV/Olig001 capsid serotype addresses this unmet need by virtue of an ability to promote oligodendroglial expression without the need for lineage-restricted promoters. The present study extends previous observations of inherent oligotropism of AAV/Olig001 neonatal nur7 mice^4^ by showing that this tropism persists in adult brains of acutely symptomatic animals of the same strain, despite using a constitutive hybrid chicken β-actin promoter (CBh) promoter active in all cell lineages. The potential benefits of this native tropism were explored by attempting to reconstitute ASPA function in the acutely symptomatic adult nur7 brain. Although the efficacy data generated are quite preliminary in nature, the head-to-head comparison with an AAV9 vector is informative. AAV9 is distinguished from AAV/Olig001 in this study by coat protein alone. Both serotypes contained identical ASPA expression cassettes, including transgene, promoter, and poly(A) sequence, and both were self-complimentary systems, able to package pseudo double-stranded genomes. The unique coat protein point of difference appears to be sufficient to alter tropism, with the present study showing the two vectors with contrasting astroglial and oligodendroglial tropism (Figures 5A and 5B). Although the current tropism comparison is far from comprehensive, the astrogliotropic properties of AAV9 have been shown effective at rescuing the Canavan phenotype in other mouse models of disease.^14^ While this astroglial targeting appears sufficient to improve phenotype, the weight of available data supports ASPA as an oligodendrocyte-specific protein in the brain.^17^^,^^18^ Therefore it is reasonable to ask if sufficient is necessarily better. The current study shows clear advantages of AAV/Olig001 for gene replacement of ASPA function in nur7 mice, with this serotype showing superior dose-responsive improvements in both motor function (Figure 5C) and gross histopathology (Figure 7). These improvements could not be attributed to increased levels of transgene expression, as AAV9 in this study was clearly more efficient in transducing vgs in the nur7 brain (Figure 6A). Unfortunately, commercially available antibodies were unable to reliably detect ASPA transgene-positive cells *in vivo*, precluding a side-by-side analysis of transgene distribution and tropism of AAV/Olig001-ASPA and AAV9-ASPA. It is possible that AAV9 in the microenvironment of the nur7 brain exhibits unexpectedly high oligotropism, but published data using the vector in other Canavan mouse models render this possibility unlikely.^14^^,^^19^ It would also be reasonable to ask if the use of Olig2 as a marker of oligodendrocytes exclusively is not in fact subject to misinterpretation given that Olig2 can also be detected in developing white matter tract astrocytes,^20^ meaning the transduction of 31% of the resident nur7 SCWM oligodendrocytes reported here (Table 3) may overstate the role of oligotropism in observed improvements over AAV9. Resolution of this potential confound would require use of an alternative lineage marker that would detect positive soma for stereological quantification.

One important observation was the fact that the cerebellum appeared refractory to high levels of oligodendroglial transduction by AAV/Olig001. Although all other ROAs employed to deliver AAV/Olig001 to 6-week-old nur7 mice resulted in high levels of oligotropism (cortex: 60%–75%, SCWM: 90%–95%, striatum: 82%–85%), transduction of the cerebellum was primarily neuronal for all ROAs. Only 14%–16% of all GFP-positive cells in the cerebellum transduced by all ROAs were identified as oligodendrocytes, with large Purkinje cells accounting for the bulk of positive soma observed. It is likely that the AAV/Olig001 capsid has a natural affinity for Purkinje cells or is at least bound in significant numbers to these cells by virtue of their relatively greater surface area, being one of the largest cells in the mammalian brain. This is somewhat problematic for a technology selected primarily on the basis of its innate oligotropism and should be considered for potential off-target effects in applications where oligodendrocyte-restricted expression is desired, and it should be noted that the current study employed a constitutive CBh, expected to be active in all cell types.^21^ This being said, AAV/Olig001 was clearly able to rescue some important phenotypic markers of disease in nur7 mice, and the ability to do so being ascribable solely to a threshold level of oligodendroglial transduction is still not fully answered. Although transgene expression from AAV/Olig001 appears modest in the thalamus, midbrain, and cerebellum, areas of severe vacuolation in nur7 mice (Figure 7A), it was still able to improve profoundly gross pathology in these regions and to a significantly greater degree than seen with AAV9 (Figure 7C). Chronically elevated NAA in Canavan disease is proposed to directly cause gross pathology and vacuolation, with NAA synthase knockdown appearing a viable means to rescue this.^22^^,^^23^ Given that NAA is naturally exported to oligodendrocytes, it stands to reason that these cells will be a significant reservoir for pathologically elevated NAA and that increased oligodendroglial transduction with ASPA may have proportionately greater benefit than greater levels of transduction in nonoligodendroglial lineages. This preposition is somewhat supported by the observed relatively greater decrease in NAA in nur7 mice treated with AAV/Olig001-ASPA, despite being 3-fold less efficient than AAV9-ASPA in transducing its recombinant genome (Figure 5), suggesting targeting oligodendrocytes may be the most efficient and tolerable strategy for Canavan disease. This aim may be promoted by combining the innate oligodendrotropic properties of AAV/Olig001 with a lineage-restricted promoter. von Jonquieres et al.^24^ recently published a very nice study demonstrating the ability of a short ∼300-bp fragment of the myelin-associated glycoprotein promoter to drive high levels of transgene expression in a chimeric AAV1/2 vector without occupying an impractical proportion of available packaging space, a vital consideration when designing self-complimentary vectors. The use of such an element in AAV/Olig001 may result in a lower threshold of effective transgene expression for white matter diseases, thereby avoiding saturating the nervous system with vast quantities of vector. A refined vector system of this nature would combine targeted vector and expression elements to provide a technology that could be both more specific and tolerable.

## Materials and methods

### Animals

Nur7 mice were maintained in-house at the Association for Assessment and Accreditation of Laboratory Animal Care (AAALAC) accredited Rowan University School of Osteopathic Medicine (Rowan SOM) Animal Facility in a specific pathogen-free (SPF) room. Founder animals originated from a commercial source (Jackson Laboratory). The origins and phenotype of nur7 mice have been described elsewhere.^11^ Homozygous nur7 mutant animals were generated from the pairing of heterozygous dams and sires and identified using a custom SNP assay and real-time qPCR. Wild-type littermates were used as reference controls wherever appropriate. All animals maintained at the Rowan SOM Animal Facility were group housed and granted ad libitum access to food and water, and all procedures involving animals were conducted under approved institutional guidelines.

Rotarod analysis was performed on old male and female animals at 10, 14, 18, and 22 weeks of age (i.e., 4 weeks postvector infusion surgeries. Wild-type reference controls were drawn from littermates of nur7 mutant mice. All treatment and control cohorts were n = 12 (50/50 gender split). Rotarod testing involved two pre-test trial days of 3× runs of 5 min (60 s between each run) on an accelerating rod and a third test day where the average of 3×, 5 min runs were scored for each animal. Pairwise comparisons of group means for each individual age point were tested for significant differences by Student’s t test, and within-sample comparisons by repeated-measures ANOVA over all ages’ testing was performed.

### Surgeries

Administration of recombinant viral vector was performed by direct injection of 5 μL of test article diluted to the appropriate concentration in 0.9% saline. Concentration of vector was defined as total numbers of viral vgs, determined by qPCR quantification of DNase-resistant AAV inverted terminal repeat (ITR) sequence in the stock preparation. All ROAs involved survival surgeries performed under inhalation anesthesia (4% induction and maintenance titered to effect). All animals in this study were 6 weeks of age at the time of dosing. 1 × 10^10^, 1 × 10^11^, or 5 × 10^11^ total vgs were delivered via 4 distinct ROAs: IT, IP, i.c.v., and ICM. IT administration involved injection into the subarachnoid space with a 33G needle between L5 and L6, with a 5-μL volume manually injected for each dose. IP administration involved 5 separate 1 μL infusions to the anterior and posterior cingulum and the cerebellum (1× each hemisphere for anterior and posterior cingulum + 1× in the cerebellum) with a 33G needle and test article infused at a rate of 0.1 μL/min using a digital pump. i.c.v. administration was carried out by a 2.5-μL volume injected at 0.1 μL/min into each lateral ventricle in each hemisphere. ICM delivery required the dorsal aspect of the skull shaved and swabbed. The head was tilted slightly to form an angle of 120° to the body and a 1-cm incision made to the occipital crest. A 33G needle was inserted into the cisterna magna (CM) and 5 μL of test article delivered at a rate of 0.1 μL/min.

### BrdU labeling

Groups of naive 2-week- and 8-week-old wild-type and nur7 mice were given systemic BrdU (50 mg/kg intraperitoneally [i.p.]), twice a day for 2 consecutive days and then sacrificed on the third day. BrdU was administered at a concentration of 50 mg/kg to animals. Brain-tissue sections were processed for BrdU staining after DNA hydrolysis in 1 M HCl using a commercially available antibody (Millipore, Sigma).

### Vector production

AAV/Olig001-GFP, AAV/Olig001-ASPA, and AAV9-ASPA vectors were produced by Bamboo therapeutics (Chapel Hill, NC, USA). Details of AAV/Olig001 capsid properties are described elsewhere.^3^ Vector was produced by the transient transfection of HEK293 cells, followed by iodixanol gradient centrifugation and ion-exchange chromatography, as previously described.^25^ All vectors were self complimentary^26^ and all contained identical expression cassettes driven by a shortened CBh^21^ and an identical BGH poly(A) sequence. Both AAV/Olig001-ASPA and AAV9-ASPA contained an identical codon-optimized, 941-bp human *ASPA* CDS.

### Immunohistochemical quantification

Estimates of a GFP-positive cell number in all dose and ROA cohorts were generated by unbiased stereology using the optical fractionator method.^27^ Serial 40 μm sagittal sections were collected for each brain in a nonrandom systematic fashion, and every 4th section in each series was processed for GFP immunohistochemistry using a commercially available anti-GFP antibody (Millipore, Sigma). A total of 60 sections per brain were analyzed, spanning 1.2 mm laterally on either side of the midline. Stereology software (Stereologer; Stereology Resource Center), coupled to an upright bright-field microscope fitted with a motorized stage, was used to generate counts of GFP-positive soma within 4 different ROIs, namely, the cerebral cortex, SCWM of the corpus callosum and external capsule, striatum, and cerebellum. GFP-positive cells in the sampling fraction were converted to absolute estimates throughout each ROI using the following formula: ∑Q ∗t/h∗1/asf∗1/ssf, where ∑Q = sum of GFP-positive cells counted, t = section thickness, h = counting frame height, asf = area sampling fraction, and ssf = section sampling fraction. Vacuolation in H&E-stained sections, generated using an identical sampling methodology, was quantified as a 3-dimensional object (vacuole) volume fraction using unbiased stereology and the formula Σ*P*_*obj*_*/*Σ*P*_*re*_*f* = *A*_*obj*_*/A*_*ref*_ = *V*_*obj*_*/V*_*ref*_, where *P*_*obj*_ = region points interacting with plaques, *V*_*obj*_ = region points interacting with the reference space, *A* = area, and *V* = volume.

A 15% threshold for the CE of intrasample variance contribution to total variance (coefficient of variation [CV]) was adhered to in order to reduce technical noise masking true biological variance between individual brains and controlling for the relative SD of individual datasets. Significant differences in stereological estimates between treatment cohorts were determined using a Student’s t test, with a threshold of α = 0.05. Stereological estimates were generated by observers blinded to treatment.

Colabeling studies for GFP localization with oligodendrocytes and neurons employed native GFP transgene fluorescence and immunofluorescent labeling of Olig2 and NeuN. 40 μm sections taken from the same series as used for GFP immunohistochemistry and the generation of stereological estimates of N for GFP-positive soma were used for immunofluorescent labeling of Olig2 and NeuN. Every 4th section in a series of 32 total sections was processed for Olig2 and NeuN staining (8 processed sections per brain), and multichannel Z stacks within in each ROA of processed sections generated by confocal microscopy using software (Nikon NS-Elements) paired to a laser-scanning confocal microscope. ROIs in individual sections were outlined by software and individual points placed every 200 μm^2^ sampled at high magnification to score for both GFP immunofluorescent soma and GFP/Olig2- or NeuN-positive cell bodies. The total number of GFP-positive soma colabeling with either Olig2 or NeuN was calculated by dividing the number of GFP-positive soma by lineage-specific colabeling in each series of sections.

### Quantification of genome copy number

Numbers of recombinant vgs in transduced animals were performed by real-time qPCR using an in-house-designed and -validated TaqMan probe/primer set targeted to the BGH poly(A) sequence of vgs. All of AAV/Olig001-GFP, AAV/Olig001-ASPA, and AAV9-ASPA contained an identical BGH poly(A) sequence. A relative standard curve method was used to calculated vg copies in DNA isolated from tissues against a purified plasmid standard of known copy number. DNA was obtained from tissues using commercially available DNA isolation kits (QIAGEN). For the analysis of whole brain, DNA from one entire hemisphere was isolated (the remaining hemisphere was used for subsequent high-performance liquid chromatography (HPLC) analysis of NAA). For the analysis of discrete ROIs within brains, relevant regions were dissected out from fresh, frozen brains. Off-target organ analysis of copy number was performed on punches of fresh tissue. For all vg copy number data, mean copy number per milligram of wet tissue weight was calculated and presented.

### Western blotting

One entire hemisphere was processed for detection of ASPA transgene in AAV/Olig001- and AAV9-treated brains at 22 weeks of age. Tissue was mechanically dissociated and lysed by sonication in radioimmunoprecipitation assay (RIPA) buffer. 30 μg of total protein was analyzed by western blot using a commercial polyclonal primary antibody to ASPA (anti-ASPA/nur7; Millipore, Sigma) at 1:500 dilution.

### HPLC

An ion-paired UV detection method was used, as previously described.^12^ Frozen brains were homogenized in an acetonitrile K_2_HPO_4_ (10 mM) precipitation solution (3:1 v/w), extracted twice with chloroform, and stored at −80°C until analyzed. 50 μL of each sample was run on a Thermo Scientific HPLC system equipped with a Surveyor PDA plus UV detector and a Hypersil BDS-C18 column (5 μm particle size; 25 cm × 4.9 mm) and analyzed with ChromQuest software (Thermo Scientific). Target NAA in samples was quantified against a purified reference standard and presented as a molar quantity per gram of wet tissue weight.
